# Relationship between FGF 23, SDMA, Urea, Creatinine and Phosphate in Relation to Feline Chronic Kidney Disease

**DOI:** 10.3390/ani12172247

**Published:** 2022-08-31

**Authors:** Simona Grelová, Martina Karasová, Csilla Tóthová, Terézia Kisková, Darina Baranová, Branislav Lukáč, Mária Fialkovičová, Alena Micháľová, Lukáš Kunay, Miroslav Svoboda

**Affiliations:** 1 Small Animal Clinic, University of Veterinary Medicine and Pharmacy, 04001 Košice, Slovakia; 2 Clinic of Ruminants, University of Veterinary Medicine and Pharmacy, 04001 Košice, Slovakia; 3 Faculty of Science, University of Pavol Jozef Šafárik, 04154 Košice, Slovakia

**Keywords:** chronic kidney disease, cat, fibroblast growth factor 23, symmetric dimethylarginine

## Abstract

**Simple Summary:**

Chronic kidney disease (CKD) is the most frequent metabolic disorder affecting geriatric cats, and is a major cause of mortality among this species. Due to its progressive nature and non-specific clinical signs, an effort is being made to discover new, more efficient biomarkers of glomerular filtration for the timely diagnosis of renal insufficiency. Symmetric dimethylarginine (SDMA) and fibroblast growth factor 23 (FGF 23) are promising new markers currently of interest in veterinary medicine. This study discusses the relationship between SDMA, FGF 23 and previously used indicators of kidney function, mainly creatinine, urea and phosphate, to establish their efficacy in a clinical setting. An increase in FGF 23 in CKD patients compared to healthy cats has been recognized, despite the lack of an evident correlation of FGF 23 with other analyzed markers.

**Abstract:**

Chronic kidney disease (CKD) is a common diagnosis in older cats, and its prevalence increases with age. Conventional indirect biomarkers of glomerular filtration rate (GFR) have their limitations, and are not efficient in detecting early decreases in glomerular filtration rate. Recently, symmetric dimethylarginine (SDMA) concentrations have been proposed as a novel biomarker of GFR for the early detection of CKD. This study discusses the relationship between SDMA, FGF 23 and previously used indicators of kidney function, mainly creatinine, urea and phosphate. Ninety-nine cats were included in this study. Based on their SDMA values, 48 cats had CKD and the remaining 51 cats were used as a healthy control group. Serum of these cats was assayed for creatinine, urea and phosphate concentrations as well as FGF 23 values, and correlations between them were evaluated. Cats with CKD had higher FGF 23 concentrations than healthy cats, and no correlation was found between FGF 23 and SDMA, nor between FGF 23 and phosphate. On the other hand, phosphate strongly correlated with SDMA, urea and creatinine, making it a possible independent factor of CKD progression.

## 1. Introduction

Chronic kidney disease (CKD) continues to be one of the most common biochemical abnormalities diagnosed in cats in small animal practice. CKD is progressive in nature, and its prevalence increases with age, with up to 80% of cats over the age of 15 years being affected [1]. The reduction in renal function in affected patients reflects the severity of clinical signs. The most prominent clinical signs that necessitate a veterinary visit are related to the gastrointestinal tract (e.g., anorexia, nausea, vomiting, melena, halitosis, diarrhea). Other non-specific clinical signs include polyuria and polydipsia, muscle wasting, weight loss, lethargy, weakness and urinary incontinence. Although no treatment to date can correct the existing irreversible renal lesions of CKD, the clinical and biochemical consequences of reduced renal function can often be alleviated by supportive and symptomatic treatment. In addition, the spontaneously progressive course of CKD can be slowed by early therapeutic intervention [2]. Indirect markers of glomerular filtration rate (GFR), such as serum creatinine and urea, are currently used for diagnosis, each with its own limitations [3]. Urine protein to creatinine ratio, urine specific gravity and blood pressure values together also aid in the diagnosis and staging of CKD. In recent years, a novel biomarker, symmetric dimethylarginine (SDMA), has been utilized for the indirect measurement of GFR. SDMA is a stable molecule that originates from intracellular proteins that play a major role in basic cellular metabolism. SDMA and related compounds are provided in the nucleus of all cells, and their formation occurs by the obligatory post-transcriptional modification and methylation of arginine residues of various proteins and subsequent proteolysis [4]. Extrarenal influences, especially the influence of muscle mass, are a limitation for the use of serum creatinine in the early diagnosis of CKD. Evidence exists showing that, unlike creatinine, SDMA is not affected by muscle mass, and does not show a correlation with body weight in adult dogs or geriatric cats [5,6]. To date, available studies suggest that plasma or serum concentrations of SDMA increase with decreasing GFR, without being affected by extra-renal factors [5,6,7,8]. The International Renal Interest Society (IRIS) modified its guidelines to incorporate persistently increased SDMA values as an indicator of decreased renal function in early stages of renal disease [9].

Chronic kidney disease–mineral and bone disorder is a systemic disorder affecting hormonal and mineral metabolism due to a disturbance in calcium-phosphate homeostasis occurring as a result of the loss of renal phosphate excretion capacity [10]. Loss of functioning nephrons resulting in a reduction in GFR leads to decreased plasma phosphate excretion. In the early stages of CKD, a “trade-off” mechanism maintains plasma phosphate concentrations within physiological limits by an increase in the production of parathormone (PTH) and fibroblast growth factor 23 (FGF 23), increasing renal phosphate clearance [11]. Fibroblast growth factor 23 is a phosphaturic hormone produced by osteocytes [12] and osteoblasts [13,14] in response to hyperphosphatemia [15]. FGF 23 maintains serum phosphate levels within the normal range by increasing renal phosphate excretion and decreasing the synthesis and degradation of calcitriol, thereby reducing intestinal phosphate absorption. In addition, FGF 23 acts on the parathyroid gland and reduces PTH synthesis and secretion [16]. FGF interacts with one of a family of four FGF receptors (FGFRs) that belong to the type I transmembrane phosphotyrosine kinase receptors. FGF 23 binds to FGF receptor 1 with its coreceptor αKlotho, a single-pass transmembrane protein with homology to β-glucuronidase that is required for receptor activation mediated by FGF 23 [17]. The renal expression of αKlotho is thought to be reduced in CKD, thereby reducing the phosphaturic effects of FGF 23 [18]. Continuous loss of renal mass combined with lower Klotho expression induces resistance to FGF 23 action, thus reducing phosphaturia and resulting in phosphate retention. To maintain normophosphatemia, osteocytes continuously synthesize FGF 23. On the other hand, FGF 23 down-regulates PTH synthesis. FGF 23 binds to the Klotho–FGFR1c complex in the parathyroid glands, and thus reduces the expression of the PTH gene [19]. However, with the progression of CKD, the Klotho–FGFR1c complex decreases in the hyperplastic parathyroid glands of uremic individuals, and the feedback loop between PTH and FGF 23 is disrupted [20]. Despite high blood levels of these phosphaturic hormones, urinary phosphate excretion is reduced due to the large loss of renal mass in the advanced stages of CKD, leading to phosphate retention and hyperphosphatemia. Altogether, FGF 23 is primarily cleared by the kidneys, and in humans is thought to be a marker of GFR [21] and a predictor of renal disease progression. In cats, FGF 23 is thought to increase with the progression of CKD, and is elevated in patients with hyperphosphatemia [22].

## 2. Materials and Methods

### 2.1. Case Selection

Feline patients recruited for this study were selected from cats examined at the University Veterinary Hospital, University of Veterinary Medicine and Pharmacy in Košice, Slovakia throughout the course of one year. All the cats seen at the practice came for routine wellness screening and blood collection, regardless of their health status. All patients over 10 years of age were included. Based on IRIS recommendations, SDMA was measured for each patient. Cats with no previous history of acute kidney injury, exposure to nephrotoxic drugs or toxins, evident signs of renal disease (progressive weight loss, polyuria/polydipsia, anorexia, vomiting), or imaging abnormalities, and with normal serum biochemistry and SDMA levels within the reference range were classified as clinically healthy. Diagnosis of CKD was based on history, serum biochemistry abnormalities and signs of kidney insufficiency and/or imaging abnormalities. Cats were classified as having CKD if they exhibited at least three of the six symptoms of weight loss, poor hair quality, polyuria/polydipsia, anorexia, vomiting and radiographic kidney changes, and had concurrent azotemia or persistently increased SDMA values (SDMA > 14). Animals treated for hypertension, hyperthyroidism or diabetes mellitus were excluded from this study.

### 2.2. Blood Sampling and Evaluation

Biochemical analyses were performed on the day of admission for each cat that showed signs of dehydration after stabilizing with intravenous crystalloid fluids. Blood sampling was performed at the clinic by venipuncture of cephalic or jugular vein with minimal restraint to reduce stress. At least 2 mL of blood was collected into tubes without anticoagulant. After centrifugation, in-house biochemical analysis was performed using a Cobas c 111 analyzer (Roche, Switzerland, CH) and Catalyst One (IDEXX Laboratories, Westbrook, ME, USA) for SDMA analysis. Remaining serum was stored at −80 °C for future analysis. These samples were later used for the measurement of intact FGF 23 by validated enzyme-linked immunosorbent assay (Cat Fibroblast Growth Factor 23 ELISA Kit, MyBioSource Inc., San Diego, CA, USA) by an automated chemistry analyzer Alizé (Lisabio, Poully en Auxois, France). Serum samples (*n* = 12) were measured using a feline FGF 23 ELISA kit to determine the coefficient of variation (CV) with low, medium and high FGF 23. From the 12 samples, the highest and lowest values were excluded, leaving 10 samples for further assessment. We measured each sample for FGF 23 5 times for intra-assay CV. The lowest limit of detection of the assay was assessed by confirmation of a mean CV < 15% for the lowest sample measured repeatedly. The effect of storage and temperature stability was assessed by storing 5 samples with baseline values measured before storage at 4 °C, −20 °C and −80 °C (frozen for 4 h and 7 days, respectively).

### 2.3. Statistical Analysis

Correlograms were performed using programming language R (version 3.6.0, accessed on 5 May 2022) with standard library and libraries ggplot2 (version 3.1.1, accessed on 5 May 2022), ggpubr (version 0.2, accessed on 5 May 2022), psych (version 1.8.12, accessed on 5 May 2022) and GGally (version 1.4.0, accessed on 5 May 2022). Statistical analyses were performed using GraphPad Prism 8.0. (GraphPad Software, Inc., San Diego, CA, USA). All data were examined for normal distribution and analyzed using the Mann–Whitney U test. The data from all animals were used; no animal was excluded for the analysis. The data are presented as median ± standard deviation (SD).

## 3. Results

Between September 2020 and August 2021, 108 cats over the age of 10 years were identified as suitable for the study, of which 9 were excluded before analysis due to sample impairment. Overall, 99 geriatric cats (42 females and 57 males) were included in the analysis, and their age ranged from 10 to 22 years (12.6 ± 2.4). The most prevalent breeds were domestic shorthair (*n* = 69), British shorthair (*n* = 11), Persian (*n* = 9), ragdoll (*n* = 5), Maine coon (*n* = 3), and additionally there was one Siamese and one Egyptian mau. All 99 cats had their SDMA measured on the day of admission, and 48 cats had increased serum SDMA concentrations. The remaining 51 had SDMA concentrations within the reference range (≤14 µg/dL). Outliers were identified: two cats with SDMA concentration 85 µg/dL and 75 µg/dL, respectively. Analysis was repeated without the outliers without any difference in the results.

**Figure 1:**
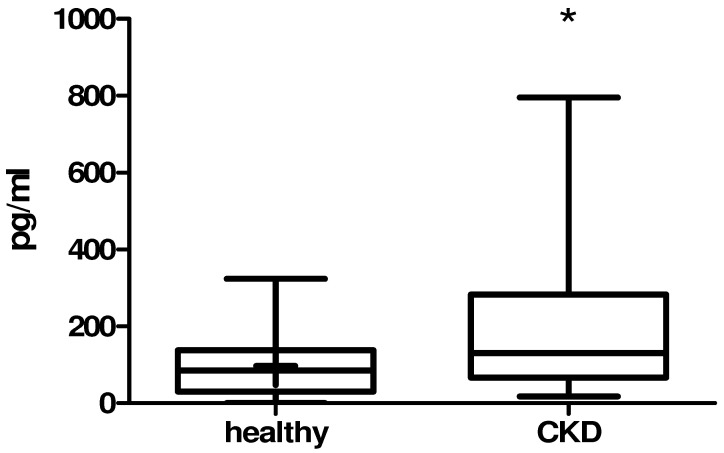
Comparison of FGF 23 between healthy and CKD cats. Data are expressed as median ± SD. The data were analyzed using the Mann–Whitney U test. The box indicates the lower and upper quartile, and the central line is the median. The points at the ends of the whiskers are the 2.5 and 97.5% values. The box plot shows the serum level of FGF 23 in healthy and CKD cats. The FGF 23 level of CKD animals is significantly higher than in healthy controls. Significance vs. healthy animals is given as * *p* < 0.05.

Results of the laboratory analysis are summarized in Table 1. The analysis revealed an increase in all of the parameters in the CKD group compared to the healthy control group of cats. In cats with CKD, serum FGF 23 concentration was significantly higher compared to the control group (Figure 1).

**Figure 2:**
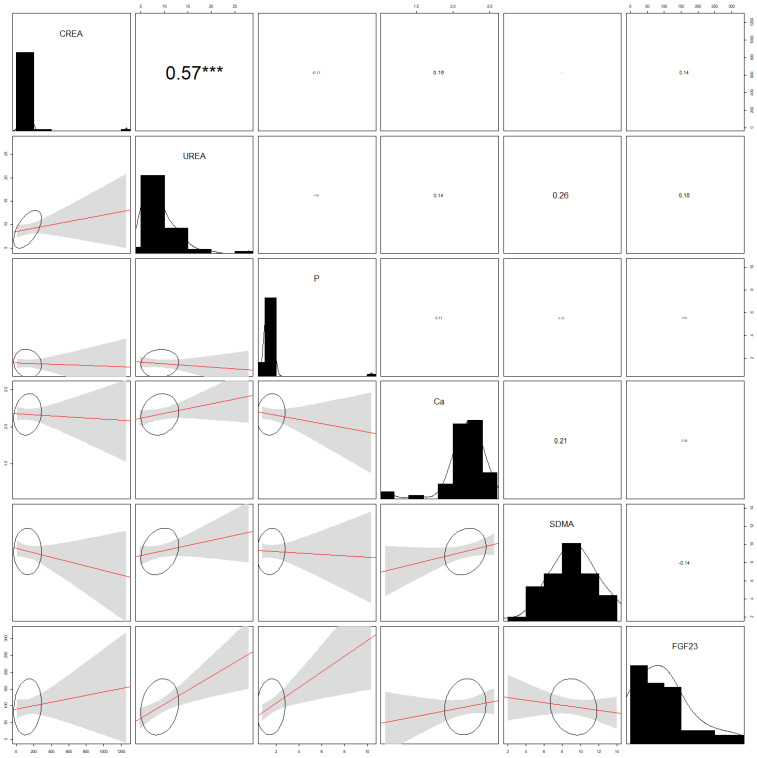
Analysis of correlations (correlograms) among levels of selected parameters in healthy cats. CREA, creatinine; P, phosphate; Ca, calcium; SDMA, symmetric dimethylarginine; FGF 23, fibroblast growth factor 23. The coefficients of Pearson product-moment correlation are reported. Significance is given by *** *p* < 0.001.

**Figure 3:**
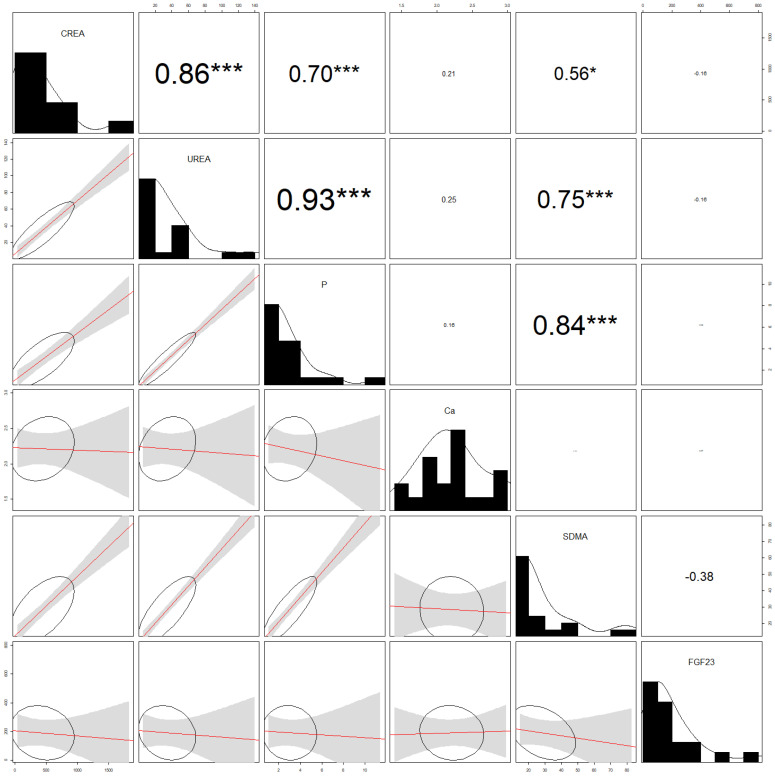
Analysis of correlations (correlograms) among levels of selected parameters in the CKD group of cats. CREA, creatinine; P, phosphate; Ca, calcium; SDMA, symmetric dimethylarginine; FGF 23, fibroblast growth factor 23. The coefficients of Pearson product-moment correlation are reported. Significance is given by * *p* < 0.05 and *** *p* < 0.001.

Correlations are shown in Figure 2 for the healthy control group and in Figure 3 for the CKD cats. Serum creatinine and urea showed a positive correlation (r^2^ = 0.57; *p* ˂ 0.001) in healthy (Figure 2) and CKD (Figure 3) cats (r^2^ = 0.86; *p* ˂ 0.001). Moreover, in the CKD group (Figure 3), significant positive correlations were also found between serum creatinine and phosphate (r^2^ = 0.70; *p* ˂ 0.001), and between urea and phosphate (r^2^ = 0.93; *p* ˂ 0.001). Additionally, in the CDK group of cats, SDMA positively correlated with phosphate (r^2^ = 0.84; *p* ˂ 0.001) and urea (r^2^ = 0.75; *p* ˂ 0.001). In addition, in the CDK group, a positive correlation was also found between creatinine and SDMA (r^2^ = 0.56; *p* ˂ 0.05).

## 4. Discussion

The aim of our study was to evaluate the relationship between SDMA, FGF 23, creatinine, urea and phosphate in geriatric cats with CKD. Because previously published studies [22,23,24,25,26] came with the hypothesis that FGF 23 should increase with increasing SDMA and should be positively correlated to phosphate concentrations, we also evaluated these two parameters. Studies indicate that FGF 23 increases with the progression of CKD in humans, dogs and cats [22,23,24,25], and therefore could be a biomarker for the early detection of CKD [27].

SDMA and serum creatinine are both considered indirect biomarkers of GFR, which was also established in our study by a positive correlation between these two parameters in the CKD group, but not the healthy group. SDMA is an endogenous molecule originating as a by-product of cellular catabolism. It is excreted primarily by glomerular filtration [5], with about 90% of the produced SDMA being eliminated by the kidneys [28]. In cats with CKD, serum SDMA increases with reduced GFR [5] and is a more effective indicator of early decrease in renal function [8]. Moreover, it is not affected by age, and in contrast to creatinine, SDMA concentrations increase with age as the GFR declines [8].

Cats in our study were grouped according to their SDMA concentrations into two groups: one consisted of healthy cats and the second group consisted of cats with CKD to allow for comparison between non-azotemic healthy cats and cats with evidence of renal disease. As with many previous studies, we also found that FGF 23 increases in cats with CKD as opposed to healthy non-azotemic cats. A negative correlation between FGF 23 and GFR was previously established in cats with CKD [29]. Due to the retrospective nature of our study, we have no data concerning GFR for selected patients. Studies in human medicine show that SDMA is a more accurate estimate of GFR and a more specific marker of kidney function than creatinine [30].

In our study, there was no significant correlation between FGF 23 and phosphate in the CKD group. However, we found a positive correlation between SDMA and serum phosphate, indicating that a disturbance in phosphate metabolism may take place early in the disease.

Hyperphosphatemia has been associated with the progression of renal disease in human patients with CKD, as well as shorter survival time in cats [31], and phosphate-restricted diets resulted in improved survival [32,33]. Since phosphate is freely filtered at the glomerulus, its increase in CKD can be associated with a decline in GFR, making it a marker of CKD progression. We found a significant positive correlation between phosphate and serum urea, and between creatinine and SDMA, suggesting that phosphate concentrations could be used as an independent predictor of CKD progression. Previous studies suggest that increasing severity and frequency of hyperphosphatemia may indicate more serious CKD [34] and shorter patient survival time [35]. Serum phosphorus concentrations result from a net balance between dietary intake and the renal excretion of phosphorus. Therefore, the maintenance of serum phosphorus concentrations in the normal range when renal function declines requires an adjustment of phosphorus intake. Phosphorus restriction is one of the main characteristics of the renal diet. Because protein is the main source of phosphorus, phosphorus-restricted diets are usually also protein-restricted. Phosphorus content is reduced to limit phosphorus retention, hyperphosphatemia, secondary renal hyperparathyroidism and the progression of renal disease. Although phosphorus retention and hyperphosphatemia are unlikely to directly cause clinical symptoms, they may promote elevated FGF 23 levels, secondary renal hyperparathyroidism, decreased calcitriol levels and renal mineralization that may contribute to CKD progression [2]. In cats, high dietary phosphorus intake (1.56% phosphorus in dry matter) for 65 to 343 days was associated with kidney mineralization, fibrosis and mononuclear cell infiltration, unlike lower phosphorus intake (0.42% phosphorus in dry matter) [36]. Not all cats in our study were fed a low-phosphorus diet or had oral phosphate binders administered; hence, we did not monitor the effect of dietary phosphorus restriction on the progression of CKD.

In human medicine, plasma FGF 23 and PTH concentrations increase in CKD patients before the development of hyperphosphatemia [37,38]. A study carried out previously on client-owned cats over the age of 9 years demonstrated that an increase in both plasma FGF 23 and PTH was a predictor of early-onset azotemia [29,39]. Rodent models demonstrate that FGF 23 increases as a response to hyperphosphatemia [15] to inhibit phosphate reabsorption in the kidney [39]. We found no correlation between FGF 23 and serum phosphate concentrations in cats with CKD. Similarly, a study conducted by Sargent et al. found significantly higher FGF 23 concentrations despite no difference in PTH or phosphate concentrations in cats with increased SDMA [40].

We identified some potential limitations of our study. One potential limitation is that the serum FGF 23 concentration measurement was not assessed at the time of sampling. Samples were stored at approximately −80 °C for 6–12 months, which could lead to degradation, affecting the measured concentrations. The stability of FGF 23 in serum frozen at −80 °C for a longer period of time has not yet been established. However, we did not notice negative effects of storage on FGF 23 in our study. Another possible limitation of this study is that the patients were not fed a uniform, controlled diet, as this could impact FGF 23 concentrations in cats receiving phosphate-restricted renal diets as opposed to those fed commercially available feline diets or owner-prepared meals [32,33].

## 5. Conclusions

We observed that FGF 23 was higher in cats with SDMA above the reference range in comparison to healthy cats, which suggests a disturbance of phosphate metabolism in early stages of the disease before hyperphosphatemia becomes evident. However, no significant correlation between FGF 23 and phosphate was found. On the other hand, phosphate strongly correlated with urea, creatinine and SDMA, making it a possible prognostic marker for the progression of renal disease. To conclude, FGF 23 alone may not be a sufficient parameter for establishing kidney function, and standard renal parameters are still considered the gold standard for routine renal disease diagnostics.

## Figures and Tables

**Table 1:** Results of laboratory analysis of biochemical parameters.

Parameter	Reference Range	Healthy		CKD	
		Median ± SD	Min–Max	Median ± SD	Min–Max
Creatinine µmol/L	59–168 ^1^	107.80 ± 158.30	11.60–1255.00	206.80 ± 511.60 *	37.50–1833.00
BUN mmol/L	8.2–12.1 ^1^	7.82 ± 4.03	4.78–27.97	18.25 ± 34.39 *	5.52–139.7
P mmol/L	0.8–2.08 ^1^	1.34 ± 1.26	0.78–10.40	1.34 ± 2.53	1.07–11.44
Ca mmol/L	2.25–2.99 ^1^	2.22 ± 0.28	1.08–2.56	2.21 ± 0.45	1.40–2.98
SDMA µg/dL	0–14 ^2^	9.00 ± 2.58	2.00–14.00	18.00 ± 20.20 *	15.00–83.00
FGF 23 pg/mL	56–700 ^3^	5.61 ± 83.98	0.00–323.90	130.20 ± 189.20 *	18.30–795.30

^1^ Reference range generated from normal clinical values. ^2^ Reference range based on IRIS. ^3^ Reference range based on Finch et al. (2013). Data are expressed as median ± SD (standard deviation). Minimal and maximal values are available. Significance is given vs. Intact by * *p* < 0.05.

## Data Availability

All data are provided in the article.
